# Slice orientation and muscarinic acetylcholine receptor activation determine the involvement of N-methyl D-aspartate receptor subunit GluN2B in hippocampal area CA1 long-term depression

**DOI:** 10.1186/1756-6606-4-41

**Published:** 2011-11-15

**Authors:** Thomas E Bartlett, Jie Lu, Yu Tian Wang

**Affiliations:** 1 Brain Research Centre, University of British Columbia, 2211 Wesbrook Mall, Room F270, Vancouver, BC, V6T 2B5, Canada

**Keywords:** Hippocampus, Long-term depression, N-methyl D-Aspartate receptor, muscarinic acetylcholine receptor

## Abstract

**Background:**

The contribution of different GluN2 subunits of the N-methyl D-aspartate (NMDA) receptor to the induction of bidirectional hippocampal synaptic plasticity is a controversial topic. As both supporting and refuting evidence for the hypothesis of subunit specialization in opposing directions of plasticity has accumulated since it was first proposed a few years ago, we hypothesize that differences in experimental conditions may have in part contributed to some of the inconsistent results from these studies. Here we investigate the controversial hypothesis that long-term depression (LTD) is preferentially induced by GluN2B-containing NMDA receptors in area CA1 of hippocampal slices.

**Results:**

We find that brain slices from 2-3 week old rats prepared in the sagittal orientation have GluN2B-independent LTD whereas slices prepared in the coronal orientation have GluN2B-dependent LTD. There was no difference between the orientations in the fraction of the NMDAR EPSC sensitive to a GluN2B-selective antagonist, leading us to believe that the intracellular signaling properties of the NMDARs were different in the two preparations. Coronal slices had greater association of LTD-related intracellular signaling protein RasGRF1 with GluN2B relative to sagittal slices. Antagonism of muscarinic acetylcholine receptors (mAChRs) in the sagittal slices returned LTD to a GluN2B-dependent form and increased the association of GluN2B with RasGRF1.

**Conclusions:**

These results suggest a novel form of NMDAR modulation by mAChRs and clarify some disagreement in the literature.

## Background

Long term potentiation (LTP) and long-term depression (LTD) of synaptic transmission are the two best-understood mechanisms by which the functional connectivity of neurons is altered [1,2]. In many brain areas, including the most-studied CA3:CA1 synapse of the hippocampus, the induction of LTP and LTD depends on activation of N-methyl D-aspartate receptors (NMDARs). NMDARs are heterotetrameric ligand gated, Ca^2+ ^permeable ion channels comprising two GluN1 subunits and two GluN2 subunits from type 2A-2D [3]. It remains unclear whether different subunits of the NMDAR are preferentially coupled to LTP or LTD induction, however different GluN2 subunits do confer different functional properties on the NMDAR. For example the GluN1/GluN2B subtype has slower channel deactivation and greater coupling to CaMKII than the GluN1/GluN2A subtype [4,5]. Based on the prolonged Ca^2+ ^flux requirements for LTD induction [6] and the developmentally decaying profile of artificially inducible LTD [7] matching the early postnatal predominance of GluN2B expression [8], it was hypothesized that GluN2B is important for LTD induction. In accordance with this idea, the predominant extrasynaptic localization of GluN2B [9] matches the requirement for extrasynaptic NMDAR activation for the induction of LTD [10]. Indeed, LTD in GluN2B ^-/- ^mouse strains is lost [11,12]. However, the results of experiments on LTD *in-*vitro with the GluN2B-selective antagonists Ro 25-6981 (Ro) [13] and Ifenprodil have been in disagreement, with various groups reporting an enhancement of LTD at the CA3:CA1 synapse [14], no effect on LTD induction [15,16] or a complete block of LTD induction at the same synapse [17]. Outside CA1 the situation is even more complex, with reports that GluN2B is essential for LTD in the perirhinal cortex [10], but also that both GluN2A and GluN2B are required for LTD in the amygdala [18] and anterior cingulate cortex [19].

Given the number of different laboratories involved in these studies, it is very likely that some of the conflicting data may have at least in part resulted from different experimental conditions employed. To try and resolve this confusion, we studied the methodologies of two labs with opposing results from experiments with Ro and LTD in area CA1 [15,17]. This led us to test the importance of slice orientation for the GluN2B-dependence of LTD induction. Indeed we found that GluN2B-dependent LTD was a property of the coronal slice orientation and GluN2B-independent LTD was a property of the sagittal orientation. There was no significant difference in the GluN2B-containing fraction of the NMDAR EPSC between the two orientations. However, a muscarinic acetylcholine receptor (mAChR) antagonist, scopolamine, conferred Ro-sensitivity on sagittal LTD. Furthermore, scopolamine led to an increase in the association of LTD-related signaling molecule RasGRF1 with GluN2B in sagittal slices. In the basal state, coronal slices had higher GluN2B-bound RasGRF1 than sagittal slices. This data clarifies some of the existing literature on GluN2B in LTD and hints toward an important mechanism of NMDAR regulation.

## Results

### Slice orientation determines the involvement of GluN2B in LTD induction

**Figure 1:**
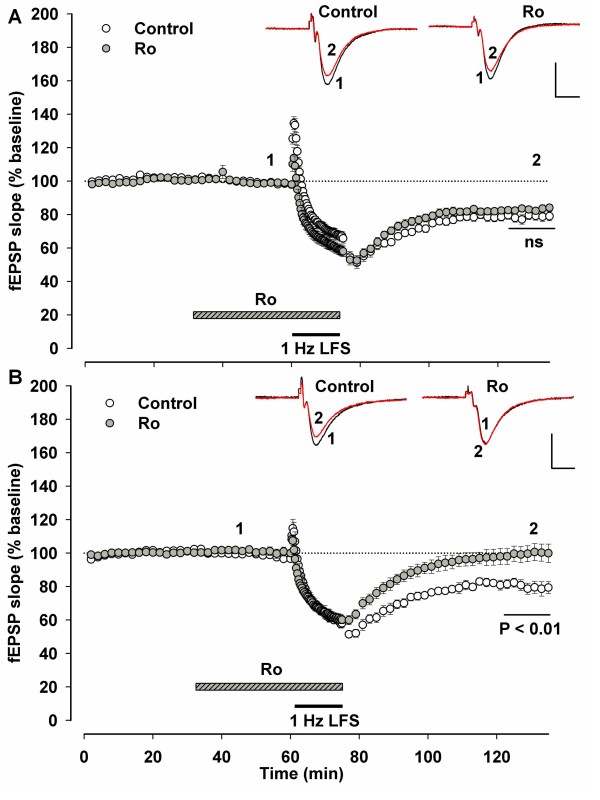
**LTD in sagittal and coronal slices has different GluN2B-dependence**. Hippocampal slices (400 μM) prepared in either sagittal (A) or coronal (B) orientations, both with CA3 attached, were used for extracellular recordings of field excitatory postsynaptic potentials (fEPSPs) in area CA1. After a stable baseline recording period (at least 30 minutes) the GluN2B-selective antagonist Ro 25-6981 (5 μM, Ro) was applied and stimulation at 0.03 Hz continued for another 30 minutes until a 1 Hz, 15 minute low frequency stimulation (LFS) at baseline intensity, also in the presence of Ro. Drug application was stopped at the end of LFS and the magnitude of LTD was quantified in the last ten minutes of the hour after LFS. In the slices of either orientation that were not treated with Ro, LFS induced LTD. Likewise, in the sagittal slices, Ro did not prevent the induction of LTD (A) although D-AP5 (50 μM) did (data not shown). However, in the coronal slices treated with Ro, LFS did not induce LTD (B). Scale bars represent 10 ms and 0.5 mV.

Faced with an apparent contradiction between the results of some groups regarding the involvement of GluN2B in LTD induction, we sought a resolution to the disagreement and an understanding of the underlying physiological mechanism. In one of the studies that found no involvement of GluN2B in LTD a sagittal [15] orientation was used when cutting the slices. In work that found LTD induction absolutely required GluN2B, the slices were prepared in a coronal orientation [17]. Thus we tested the importance of preparing the hippocampal slice in two different orientations. When we interleaved experiments preparing the slices in one of the two orientations and treating the slices with the GluN2B antagonist Ro 25-6981 (Ro), we found that the LTD induced by 1Hz low frequency stimulation (LFS) in the sagittal slices was GluN2B-independent (Figure 1A. Control, n = 13, 78.8 ± 2.66% baseline. Ro, n = 9, 80.7 ± 2.14% baseline. P = 0.619) consistent with the previous study [14], while LTD in the coronal slices was GluN2B-dependent (Figure 1B. Control, n = 7, 79.5 ± 3.62% baseline. Ro, n = 5, 100.2 ± 3.10% baseline. P = 0.002) in full agreement with the previous work [16]. LTD in the sagittal slices was completely NMDAR-dependent as shown by interleaved experiments where the pan-NMDAR antagonist 50 μM D-AP5 blocked LTD induction completely (data not shown).

**Figure 2:**
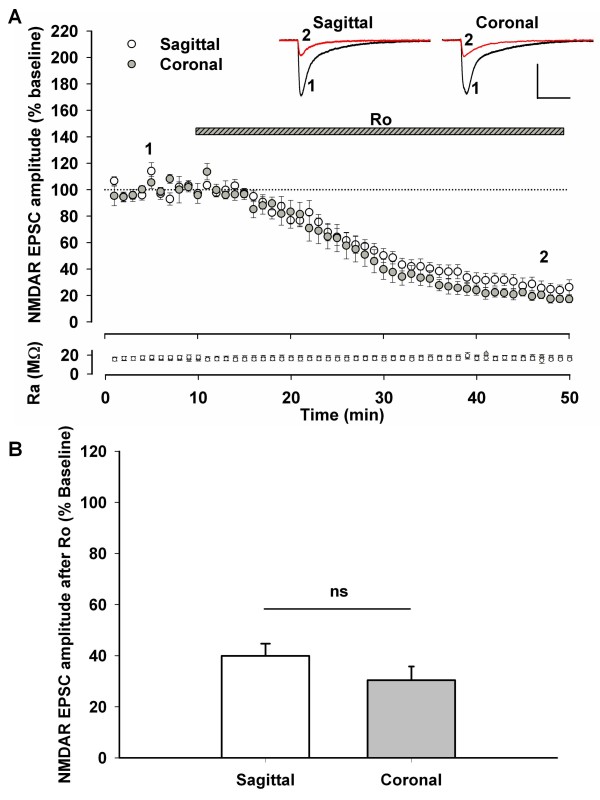
**The GluN2B-mediated fraction of the NMDAR EPSC is not significantly different between coronal and sagittal orientations**. A) Whole-cell patch clamp recordings from CA1 pyramidal cells in either sagittal or coronal slices were made and the NMDAR EPSC was isolated at -40 mV in the presence of bicuculine (10 μM) and NBQX (5 μM). After a 10 minute stable baseline recording period at stimulation frequency 0.03 Hz, the GluN2B-selective antagonist Ro was applied for 40 minutes while stimulation continued. Scale bars represent 200 ms and 200 pA. B) The level of depression in the NMDAR EPSC induced by Ro, when quantified in the 40-50 minute epoch, was similar in sagittal and coronal slices. This indicates activation of a similar number of synaptic GluN2B-containing NMDARs during baseline synaptic stimulation in either slice orientation.

The simplest explanation for the difference in the GluN2B requirements of LTD induction in the two slice orientations would be much reduced synaptic expression of GluN2B in the sagittal slices, leading to GluN2B-independent LTD. To our surprise, we found no significant difference between the effect of Ro on the NMDAR EPSC from CA1 pyramidal cells in slices prepared in the sagittal orientation versus cells from coronal slices (Figure 2, sagittal, n = 8, 39.9 ± 4.72% baseline, Coronal, n = 7, 30.4 ± 5.33% baseline, P = 0.202). The induction of LTD is dependent not only on NMDAR activation and Ca^2+ ^influx, but on the activation of a cascade of intracellular processes leading to the expression of LTD [20]. Thus we supposed that a change downstream of NMDAR activation was responsible for the altered subunit dependency of LTD in the two slice orientations.

### mAChR antagonist scopolamine increases the involvement of GluN2B in LTD induction

**Figure 3:**
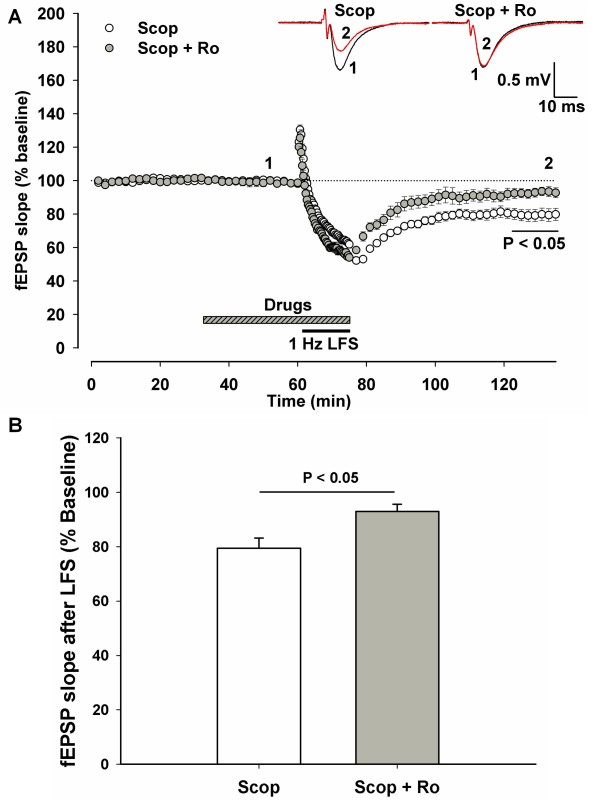
**GluN2B-dependence of LTD is created in the sagittal slice by a muscarinic acetylcholine receptor antagonist**. A) Sagittal slices were again used for extracellular recordings of synaptic activity. After a stable baseline stimulation period (0.03 Hz, >30 minutes), the muscarinic receptor antagonist scopolamine (Scop, 20 μM) was added to the aCSF with or without GluN2B-selective antagonist Ro (5 μM) and stimulation continued at baseline frequency for another 30 minutes. LFS was then begun while drug application continued. Drug washout began at the termination of LFS and the magnitude of LTD was quantified in the final 10 minutes of the hour after LFS. B) In the sagittal slices, application of scopolamine did not change the induction of LTD or the baseline fEPSP. However, when co-applied with GluN2B antagonist Ro there was a significant inhibition of LTD compared to the slices treated with scopolamine alone. Scale bars represent 10 ms and 0.5 mV.

The major cholinergic innervation to area CA1 comes via the medial septal nucleus and releases acetylcholine onto both principal cells and interneurons [21]. We proposed that the sagittal slice may retain a greater component of innervation than the coronal section [22]. Hence we tested the hypothesis that antagonism of muscarinic acetylcholine receptors (mAChRs) in the sagittal slices would recapitulate the phenotype of the coronal slices. In sagittal slices, we found that co-application of the mAChR antagonist scopolamine (20 μM) enabled block of LTD by the GluN2B antagonist Ro. (Figure 3. Scopolamine, n = 9, 79.5 ± 3.64% baseline. Ro + Scopolamine, n = 6, 93.0 ± 2.64% baseline. P = 0.018). Scopolamine alone had no significant effect on either the baseline fEPSP or the magnitude of LFS-induced LTD.

### GluN2B interaction with RasGRF1 is higher in conditions with GluN2B-dependent LTD

**Figure 4:**
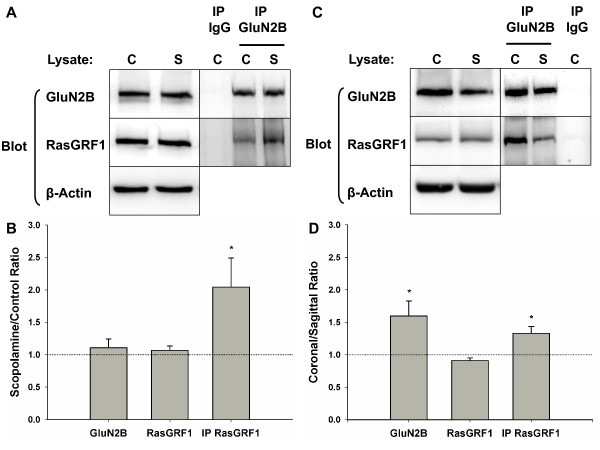
**Greater association of GluN2B with RasGRF1 occurs under conditions that show GluN2B involvement in LTD**. A,B) Sagittal hippocampal slices were prepared with the same methodology as for the electrophysiological experiments and treated with control aCSF (C) or 20 μM scopolamine in aCSF (S) for 2 hrs, followed by homogenization in RIPA buffer. 500 μg of each lysate was used for immunoprecipitation with a polyclonal GluN2B antibody bound to protein A sepharose. After washing in RIPA, the coIPs were subjected to SDS PAGE and Western blot, alongside 30 μg per lane of the crude lysates. The blots were sequentially probed with RasGRF1 and GluN2B antibodies using HRP- and AP-conjugated secondary antibodies respectively. In each coimmunoprecipitate the level of RasGRF1 was normalized to GluN2B and then a scopolamine/control ratio was calculated and averaged across experiments. C, D) Coronal (C) or sagittal (S) slices were prepared and recovered in the absence of scopolamine. The lysates were processed and taken forward into immunoprecipitation as described above.

We then asked whether the enhanced involvement of GluN2B in LTD after scopolamine treatment could be the result of an increased association with an LTD-related signaling protein. Synaptic plasticity downstream signaling can involve MAP kinase activation, most commonly p38 MAPK for LTD [23,24]. One pathway to MAP kinase signaling is the small GTPase Ras, which is active in the GTP-bound state and inactive after bound GTP is hydrolyzed to GDP. RasGRF1 is a synaptic Ras-specific guanine nucleotide exchange factor that catalyses the release of GDP from Ras and subsequent activation by GTP [25]. RasGRF1 is required for LTD induction and is a binding partner of the carboxyl-tail of GluN2B [23,26]. When we immunoprecipitated GluN2B from a lysate of hippocampal slices that had been subject to scopolamine or control treatments we found that the ratio of RasGRF1 to GluN2B in the immunoprecipitate was significantly increased in the scopolamine condition, supporting our hypothesis that increased association of RasGRF1 with GluN2B is part of the mechanism by which GluN2B can be included in LTD signaling (Figure 4A,B, Ratio of normalized RasGRF1 in precipitate scopolamine/control = 2.04 ± 0.451, P = 0.0415, n = 12). No increase in RasGRF1 or GluN2B was detected in the crude lysate from the scopolamine slices compared to control slices (Figure 4A, B, Ratio of normalized RasGRF1 in lysate: scopolamine/control = 1.067 ± 0.0674, P = 0.340, n = 12, Ratio of normalized GluN2B in lysate: scopolamine/control = 1.11 ± 0.137, P = 0.441, n = 12).

When we used recovered but untreated coronal or sagittal hippocampal slices to prepare protein lysates for the GluN2B coimmunoprecipitation we found that the immunoprecipitates from the coronal slices were significantly enriched for GluN2B-bound RasGRF1 relative to the sagittal slices (Figure 4C and 4D, coronal/sagittal ratio of normalized RasGRF1 in GluN2B immunoprecipitate = 1.330 ± 0.105, P = 0.020, n = 4). Furthermore, we also saw a greater level of GluN2B in the coronal slice lysates relative to slices prepared in the sagittal orientation but no difference in lysate RasGRF1 (Figure 4C and 4D, Ratio of normalized RasGRF1 in lysate: coronal/sagittal = 0.909 ± 0.0445, P = 0.086, n = 4, Ratio of lysate GluN2B coronal/sagittal = 1.60 ± 0.229, P = 0.04, n = 4). Previously we did not detect a change in GluN2B at the synapses of coronal versus sagittal slices using electrophysiology to measure NMDAR EPSCs (Figure 2).

## Discussion

The major findings of this paper are that the GluN2B subunit involvement in LTD induction in hippocampal area CA1 is different according to slice orientation and mAChR activation. The GluN2B-dependence of LTD in the two orientations is not a consequence of the synaptic number of GluN2B-containing receptors, but consistent with an alteration in GluN2B coupling to downstream signaling molecule RasGRF1 via a mechanism regulated by tonic mAchR activation. We show that decreased mAchR tone increases the interaction of GluN2B with RasGRF1 and the involvement of GluN2B in LTD.

The hippocampus receives cholinergic input from the medial septum which passes into all subfields including CA1 [21,27,28]. Alternate orientations of slice have been shown to change the properties of other synapses in the brain, for example, the synaptic transmission and plasticity of the amygdala is different between coronal and horizontal slices [29]. It is possible that more cholinergic fiber tracts are spared in the sagittal orientation than in the coronal orientation, thus changing the properties of LTD according to the level of basal mAChR activation. All of the mAChR subtypes (M1-M5) are present in CA1 and may contribute to the phenomenon we observe here [30,31]. M2/4 subtypes couple through Gi to a reduction in cAMP levels, whereas M1/M3 couple through Gq to a rise in intracellular Ca^2+ ^from intracellular stores. mAChRs elevate the excitability of CA1 pyramidal cells and modulate synaptic transmission in multiple ways from both a presynaptic and postsynaptic locus.

Presynaptic M2 AChRs inhibit ACh release. Scopolamine could thus facilitate ACh release if these receptors are tonically active [32], but would also inhibit postsynaptic receptors. Repetitive stimulation of the Stratum Orients (SO) has been shown to evoke a cholinergic slow EPSP that increases CA1 pyramidal cell excitability [33]. Stratum Radiatum (SR) stimulation preceded by repetitive SO stimulation delivers enhanced LTP relative to SR stimulation alone, via facilitation of NMDARs after inhibition of SK channels [34,35]. The effect of evoking extra ACh release on the subunit-dependency of LTD is unknown; however mAChRs also regulate the surface expression of NMDARs. M1 receptors activate a pathway involving intracellular Ca^2+^, hippocalcin and clathrin-dependent endocytosis of NMDARs [36]. It remains to be seen whether this pathway preferentially modulates GluN2A or GluN2B.

GluN2B-containing receptors have a predominantly extrasynaptic localization that may only be accessed by glutamate during extended stimulation protocols such as the LFS used here to induce LTD. As such, our quantification of GluN2B via the NMDAR EPSC (Figure 2) may overlook differences in the extrasynaptic NMDAR population between the slice orientations. Indeed, we observed greater GluN2B levels in the lysates of coronal slices relative to sagittal slices (Figure 4). Therefore, coronal slices may have greater involvement of GluN2B in LTD because of greater GluN2B levels relative to sagittal slices, as well as greater interaction of these NMDAR subunits with RasGRF1 in the coronal slices (Figure 4).

Diffuse, rather than synaptic, transmission by acetylcholine is suggested by the lack of co-localization of cholinergic varicosities with glutamatergic synaptic elements and the tonic baseline concentration of ACh in the extracellular space of slices [37]. Tonic extracellular ACh levels in the slice would explain the action of scopolamine, and may be determined by slice preparation conditions. Higher extracellular Ach in the sagittal slice relative to the coronal slice would be consistent with the current study. Ambient ACh would not be restricted to the synapse and could modulate mAChRs in the vicinity of extrasynaptic GluN2B-containing receptors to elicit a change in GluN2B downstream signaling.

RasGRF1 is coupled to p38 MAPK activation, required for LTD induction [23] and associates with the C-terminal domain of GluN2B [26]. The increased association of GluN2B and RasGRF1 seen in this study after scopolamine treatment of slices suggests a mechanism by which mAChR activation usually restricts the involvement of GluN2B in the signaling for LTD. When tonic activation of mAChRs is prevented in the sagittal slice by mAChR antagonism there is greater involvement of GluN2B in LTD induction (Figure 3). In the untreated condition there is greater association of GluN2B with RasGRF1 in the coronal slices relative to the sagittal slices (Figure 4), suggesting a mechanism to account for the sensitivity of coronal LTD to the GluN2B-selective antagonist Ro 25-6981.

Loss of cholinergic brain function is a factor in normal aging and is especially prominent in several neurodegenerative disorders [38,39]. Our data suggests that reduction in acetylcholine occupation of muscarinic receptors leads to increased LTD-related- and RasGRF1/Ras/MAPK signaling from GluN2B-containing NMDA receptors. Considering that GluN2B-containing, extrasynaptic NMDA receptors promote excitotoxic cell death while synaptic GluN2A-containing receptors promote cell survival [40-45], it is tempting to speculate that this mechanism is important in glutamate-receptor related pathology.

## Conclusions

We conclude that the difference between some labs doing experiments on GluN2B involvement in LTD [15-17] can be explained at least in part by slice orientation. This highlights the need for more physiologically relevant preparations such as behaving animals [46]. Furthermore, we have found that there is flexibility in the coupling of GluN2B to LTD induction, such that reduced signaling from mAChRs increases the association of GluN2B with RasGRF1 and the sensitivity of LTD to a GluN2B antagonist. Thus, the NMDAR subunit rules in regulating synaptic plasticity may be more dynamic then previously envisaged, and subject to dynamic regulation under different physiological and pathological conditions.

## Methods

### Slice preparation

Male Sprague-Dawley rats aged 14-21 days were euthanized in accordance with UBC animal care policy and the brain rapidly removed into ice-cold (2-4°C) 'slicing aCSF' comprising (in mM) NaCl 124, KCl 3, NaH_2_PO_4 _1.25, NaHCO_3 _26, Glucose 15, CaCl_2 _1, MgSO_4 _10, 310 mOsm and pH 7.35 when gassed with 95% O_2 _5% CO_2_. For sagittal slices, the brain was trimmed to give flat lateral surfaces and then hemisected along the sagittal plane before being glued onto a vibratome stage with the medial surface uppermost. For coronal slices the rostral end of the brain was chopped with a coronal cut to create a flat surface upon which to glue the brain with the caudal end uppermost. Slices (400 μM thick) were prepared from the dorsal hippocampus and recovered at room temperature (23-25°C) for at least 2 hours in 'recording' aCSF with CaCl_2 _2 mM, MgSO_4 _1 mM.

### Patch-clamp electrophysiology

Slices were superfused with room temperature (23-25°C) 'recording' aCSF flowing at 2.5 ml/minute with composition as above, except including bicuculine methobromide (10 μM) and NBQX (5 μM). Pyramidal neurons in area CA1 were patched under visual guidance with glass micropipettes of resistance 4-6 MΩ when filled with a solution containing (in mM) CsMeSO_4 _130, NaCl 8, EGTA 0.5, HEPES 10, QX-314 bromide 5, Mg.ATP 8, Na.GTP 4, pH 7.25, 295 mOsm. Data acquisition hardware was via Axon Instruments amplifier Multiclamp 700A and analog-digital converter Digidata 1322a. Data acquisition and analysis software was WinLTP, [47-49]. Cells were voltage clamped at -40 mV and the Schaffer collateral/commissural pathway was stimulated with a twisted NiCr bipolar electrode at 0.03 Hz to evoke NMDAR-mediated EPSCs. After a stable baseline for 20 minutes, the application of Ro 25-6981 began. The magnitude of Ro-induced depression of the NMDA EPSC was quantified as the average of the 30-40 minute period after beginning application of Ro. Statistical comparison between the orientations were by t-test.

### Extracellular electrophysiology

Slices were superfused with room temperature (23-25°C ) 'recording' aCSF at 2.5 ml/minute and field excitatory postsynaptic potentials (fEPSPs) were measured in stratum radiatum of CA1 using a glass micropipette of resistance 2 MΩ when filled with aCSF and data acquisition hardware/software as described above. The Schaffer Collateral/Commissural pathway was stimulated as above to evoke baseline fEPSPs at 50% of the slope at which population spikes were first visible. Baseline fEPSPs were recorded for at least 30 minutes and slices with an unstable baseline were discarded. LTD was induced by low frequency stimulation (LFS) of 900 pulses at 1Hz, baseline intensity. The magnitude of LTD was quantified as the average slope of the fEPSP in the last ten minutes of the hour after LFS. Ro 25-6981 and scopolamine were from Tocris and both dissolved in water and then aCSF just before the experiment. Drugs were applied at times indicated in the figures by the filled bars. Statistical comparisons were by t-test.

### Co-immunoprecipitation

Sagittal or coronal slices were prepared as above and left to recover at room temperature (23-25°C ) for at least 2 hours. Slices were split into coronal, sagittal, control (sagittal) and scopolamine (sagittal) groups and the latter treated with 20 μM scopolamine for 2 hrs. Slices were homogenized in ice-cold RIPA buffer comprised of (in mM unless indicated) NaCl 150, Sodium deoxycholate 0.3%, Tris 50, EDTA 1, Triton X-100 1%, SDS 0.1%, protease inhibitor cocktail (Roche), pH 7.4. All following steps were conducted at 4°C or on ice. The homogenate was triturated and cleared by centrifugation at 14,000 rpm for 30 minutes. The soluble fraction was quantified and 500 μg of protein was loaded onto protein A beads (GE) that had been incubated for 1 hr with rabbit polyclonal anti-GluN2B C-terminal (YTW lab) or with normal rabbit IgG (SantaCruz, #sc2027). Beads were incubated in protein lysate with rotation overnight before four 5 minute washes in RIPA buffer and resuspension in PBS and sample buffer.

### SDS PAGE and Western Blot

Half of each immunoprecipitation was loaded per lane of an SDS PAGE gel, alongside 30 μg per lane of crude lysate. Gels were blotted onto PVDF and probed with mouse monoclonal anti-GluN2B C-terminal (Millipore, #ab28373) or rabbit polyclonal anti-RasGRF1 (SantaCruz, #sc224). Secondary antibodies were HRP-conjugated anti-rabbit (Perkin Elmer, NEF812001), or AP-conjugated anti-mouse (Promega #S372B). Luminescent substrates were Pierce ECL substrate for HRP (#32106) or Thermo Scientific Lumi Phos WB for AP (#34150). Signals were quantified with Quantity One software (BioRad) and the level of RasGRF1 in each immunoprecipitation was normalized to the respective level of GluN2B. The ratio of scopolamine to control, or of coronal to sagittal in each experiment (n) was calculated, then averaged across the experiments and analyzed in a one sample t-test.

## List of abbreviations

EPSC: Excitatory Postsynaptic Current; fEPSP: Field Excitatory Postsynaptic Potential; LFS: Low Frequency Stimulation; LTD: Long-Term Depression; LTP: Long-Term Potentiation; mAchR: Muscarinic Acetylcholine Receptor; NMDA(R): N-methyl D-aspartate (receptor); Scop: Scopolamine; RasGRF1: Ras Guanine nucleotide Release Factor 1; Ro: Ro 25-6981 (R-(R*,S*)-α-(4-hydroxyphenyl)-β-methyl-4-(phenylmethyl)-1-piperidine propanol).

## Competing interests

The authors declare that they have no competing interests.

## Authors' contributions

TEB completed the experiments and wrote the manuscript. JL made the GluN2B antibody used for the co-immunoprecipitation. YTW helped write the manuscript and coordinated the project. All authors read and approved the final manuscript.
